# The men’s health gap: men must be included in the global health equity agenda

**DOI:** 10.2471/BLT.13.132795

**Published:** 2014-03-06

**Authors:** Peter Baker, Shari L Dworkin, Sengfah Tong, Ian Banks, Tim Shand, Gavin Yamey

**Affiliations:** a Global Action on Men’s Health, Brighton, England.; b Department of Social and Behavioral Sciences, University of California, San Francisco, United States of America (USA).; c Department of Family Medicine, Universiti Kebangsaan Malaysia, Kuala Lumpur, Malaysia.; d European Men’s Health Forum, Brussels, Belgium.; e Sonke Gender Justice, Cape Town, South Africa.; f Evidence to Policy initiative (E2Pi), Global Health Group, University of California, San Francisco, 50 Beale Street (Suite 1200), Box 1224, San Francisco, CA 94105, USA.

In most parts of the world, health outcomes among boys and men continue to be substantially worse than among girls and women, yet this gender-based disparity in health has received little national, regional or global acknowledgement or attention from health policy-makers or health-care providers. Including both women and men in efforts to reduce gender inequalities in health as part of the post-2015 sustainable development agenda would improve everyone’s health and well-being.

That men tend to be in worse health than women has now been made clear by robust evidence from various sources. The Global Burden of Disease study led by the Institute for Health Metrics and Evaluation in 2010 (GBD 2010 study) showed that throughout the period from 1970 to 2010, women had a longer life expectancy than men.^1^ Over that 40-year period, female life expectancy at birth increased from 61.2 to 73.3 years, whereas male life expectancy rose from 56.4 to 67.5 years. These figures indicate that the gap in life expectancy at birth widened between the sexes to men’s disadvantage over those 40 years.

By 2010, on the whole women were outliving men by an average of almost six years. In the region with the lowest life expectancy at birth − central sub-Saharan Africa − men were living 5.3 years less than women on average. Eastern Europe showed the biggest difference in life expectancy between men and women: women in the Russian Federation were outliving men by an average of 11.6 years. According to the *Global health 2035* report, published in the* Lancet* in 2013, in countries classified as “least developed” and “less developed” by the United Nations adult mortality fell faster among women than among men between 1992 and 2012.^2^

## Explaining the gender gap

In many societies, men generally enjoy more opportunities, privileges and power than women, yet these multiple advantages do not translate into better health outcomes. What explains this gender disparity? According to the WHO European Region’s review of the social determinants of health, chaired by Sir Michael Marmot, men’s poorer survival rates “reflect several factors – greater levels of occupational exposure to physical and chemical hazards, behaviours associated with male norms of risk-taking and adventure, health behaviour paradigms related to masculinity and the fact that men are less likely to visit a doctor when they are ill and, when they see a doctor, are less likely to report on the symptoms of disease or illness”.^3^

How much more likely to die are men than women as a result of risk-taking behaviours? In 2010, 3.14 million men − as opposed to 1.72 million women − died from causes linked to excessive alcohol use.^4^ For many men, excessive consumption of alcohol is linked to notions of masculinity. For example, a study of men in the Russian Federation showed that heavy drinking of strong spirits “elevates or maintains a man’s status in working-class social groups by facilitating access to power associated with the hegemonic ideal of the real working man”.^5^ Of 67 risk factors and risk factor clusters identified in the GBD 2010 study, 60 were responsible for more male than female deaths and the top 10 risk factors were all more common in men.^4^

In many countries, research suggests that women are more likely than men to use health services, although this disparity may reflect women’s increased use of services during their reproductive years.^6^ For example, in England in 2008 and 2009, women aged 15 to 80 years had significantly more consultations with general practitioners than men; the biggest gender gap was noted in the 20- to 44-year age group.^7^ In a Lithuanian study of middle-aged university employees, women were found to be significantly more likely than men to get regular dental check-ups.^8^

Several recent studies in Malawi, South Africa, Uganda and Zimbabwe suggest that notions of masculinity not only increase the risk of infection with the human immunodeficiency virus (HIV), but they also inhibit men from getting tested for HIV, coming to terms with their HIV-positive status, taking instructions from nurses, and engaging in health-enabling behaviours.^9^ Cornell et al. have argued that we have a “blind spot” when it comes to men and antiretroviral therapy (ART) in Africa. These researchers note, for example, that disproportionately fewer men than women access ART across Africa, that men start ART later in the disease course than women, and that men are more likely than women to interrupt treatment and be lost to follow-up.^10^

Finally, the highly gendered nature of employment in all societies translates into men being more exposed to occupationally related morbidity and mortality than women. In 2010, almost 750 000 men died from occupationally related causes, as opposed to just over 102 000 women.^4^ In Europe, 95% of fatal accidents and 76% of non-fatal accidents at the workplace are experienced by men.^11^ In the United States of America, the occupations with the highest risk of fatal occupational injury, such as mining, agriculture and fishing, employ far more men than women.^12^

## Policy silence at global health institutions

As Hawkes & Buse recently noted, the gender disparities noted earlier are not properly addressed in the health policies and programmes of the major global health institutions, including WHO.^6^ Policy-makers tend to assume that gendered approaches to health improvement are primarily or exclusively about women rather than about both sexes, a position also adopted by most national governments. To the best of our knowledge, only three countries – Australia, Brazil and Ireland – have to date attempted to address men’s burden of ill health through the adoption of national, male-centred strategies.

Compounding this neglect by policy-makers are negative stereotypes of men on the part of many health-care providers. For instance, some assume that men are largely disinterested in their health – an attitude that can, in turn, discourage men from engaging with health services.^13^ Barker et al. have noted that “health programs often view men mainly as oppressors – self-centred, disinterested, or violent – instead of as complex subjects whose behaviours are influenced by gender and sexual norms”.^14^

Any serious effort to improve public health must include attention to the health needs of both sexes and responsiveness to the differences between them. Attention to men’s and women’s health will be particularly important in tackling the global epidemic of noncommunicable diseases, which are likely to affect more men than women and to affect men at a younger age.

Taking action is not just a matter of equity; it is also a matter of economics. For example, men’s underuse of primary care services in Denmark results in their use of more expensive hospital services instead,^15^ while men’s premature mortality and morbidity cost the United States economy alone an estimated 479 billion United States dollars annually.^16^

## Policy targets and effective interventions

White et al. have argued that public and policy action to improve men’s health should have three targets.^17^ The first is schools, where stereotypes about masculinity can be challenged. The second is the promotion of men’s health and well-being in the workplace. A third crucial area for policy is to target health services and health promotion towards marginalized men, men from minority populations, men in prison populations and men who have sex with men – all of whom have a higher burden of disease and early death than other men.

Three types of intervention targeting men have emerged in recent years – outreach, partnership and gender transformation – and there is now evidence to support all three approaches. Interventions in high-income countries (e.g. Australia, the United States and countries of western Europe) have generally involved outreach efforts aimed at men in pubs and bars, sports clubs, barber shops, schools and the workplace, with a focus on weight loss, smoking cessation and other lifestyle changes. In a recent randomized controlled trial of a gender-sensitized weight loss and healthy living programme for overweight or obese male soccer fans at 13 Scottish professional soccer clubs, the intervention led to significant weight loss.^18^

A second approach involves partnering with men to improve women’s and children’s health. For example, research in Ghana has shown that child vaccination programmes designed to involve fathers (not just mothers) in decisions about their children's use of preventive health services may increase timely immunization coverage levels.^19^ Similarly, systematic reviews of studies conducted in low- and middle-income countries have shown the benefits of engaging male partners in decisions about reproductive and sexual health, including family planning.^20^

A third approach, which is being increasingly supported by evidence from randomized controlled trials and other types of studies, is to support interventions aimed at gender transformation. These aim to reshape male gender roles in ways that lead to more equitable relationships between women and men. Such interventions can increase protective sexual behaviours, prevent intimate partner violence, modify inequitable attitudes linked to gender, and reduce sexually transmitted infections.^21^

## A global men’s health movement

WHO’s Regional Office for Europe has made a bold commitment to “addressing the impact of gender on men's health and involving men in achieving gender equity in the WHO European Region through WHO programmes or direct support to Member States”.^22^ However, it is unclear what actions the office has taken to date or is planning for the future. In 2011, the European Commission published a comprehensive report, *The state of men’s health in Europe*,^11^ but an action plan based on its findings has not yet been produced.

Global, regional and national health and development agencies could certainly learn from the success of civil society groups in promoting policies that target men. For example, the South African non-profit organization Sonke Gender Justice successfully pushed the government to add interventions targeting men within South Africa’s national HIV strategic plan. The charity Men’s Health Forum (England and Wales) was instrumental in persuading the government of the United Kingdom of Great Britain and Northern Ireland to extend the national chlamydia screening programme to cover young people of both sexes rather than primarily women.

Given the robust evidence of a “men’s health gap” and the emerging evidence on how to close it, the next step is to move the issue higher up on the agenda of national governments and global health institutions without diminishing efforts to improve women’s health. A new organization, Global Action on Men’s Health, has recently been established by men’s health organizations around the world to advocate for national, regional and global public health policies that take account of men as well as women.

## Conclusion

The GBD 2010 study has, we hope, helped to raise awareness of the excess burden of morbidity and mortality in men. Concerted global action to reduce this burden could have a transformative social, health and economic impact. It is time to not only acknowledge the benefits of such action to men, but also to recognize and measure its potential benefits to women, children and society as a whole. Men’s physical illness, for example, can impair the psychological health of their female partners; when men are sick, injured or die, households and female partners suffer a loss of income.^23^ Closing the men’s health gap can benefit men, women and their children.

## References

[1] Wang H, Dwyer-Lindgren L, Lofgren KT, Rajaratnam JK, Marcus JR, Levin-Rector A, et al. Age-specific and sex-specific mortality in 187 countries, 1970–2010: a systematic analysis for the Global Burden of Disease Study 2010. Lancet. 2012;380:2071-94 23245603 10.1016/S0140-6736(12)61719-X

[2] Jamison DT, Summers LH, Alleyne G, Arrow KJ, Berkley S, Binagwaho A, et al. Global Health 2035: a world converging within a generation. Lancet. 2013;382:1898-955 24309475 10.1016/S0140-6736(13)62105-4

[3] UCL Institute of Health Equity [Internet]. Review of social determinants and the health divide in the WHO European Region: final report. Copenhagen: World Health Organization, Regional Office for Europe; 2013. Available from: http://www.instituteofhealthequity.org/projects/who-european-review [cited 2014 Feb 21].

[4] Lim S, Vos T, Flaxman AD, Danaei G, Shibuya K, Adair-Rohani H, et al. A comparative risk assessment of burden of disease and injury attributable to 67 risk factors and risk factor clusters in 21 regions, 1990-2010: a systematic analysis for the Global Burden of Disease Study 2010. Lancet. 2012;380:2224-60 23245609 10.1016/S0140-6736(12)61766-8 PMC4156511

[5] Hinote BP, Webber GR. Drinking toward manhood: masculinity and alcohol in the former USSR. Men Masc. 2012;15:292-310

[6] Hawkes S, Buse K. Gender and global health: evidence, policy, and inconvenient truths. Lancet. 2013;381:1783-7 23683645 10.1016/S0140-6736(13)60253-6

[7] Hippisley-Cox J, Vinogradova Y. Trends in consultation rates in general practice 1995/1996 to 2008/2009: analysis of the QResearch® database. Leeds: Health and Social Care Information Centre, 2009.

[8] Sakalauskienė Ž, Vehkalahti MM, Murtomaa H, Mačiulskienė V. Factors related to gender differences in toothbrushing among Lithuanian middle-aged university employees. Medicina (Kaunas). 2011;47:180-6 21822041

[9] Skovdal M, Campbell C, Madanhire C, Mupambireyi Z, Nyamukapa C, Gregson S. Masculinity as a barrier to men's use of HIV services in Zimbabwe. Global Health. 2011;7:13 21575149 10.1186/1744-8603-7-13 PMC3107786

[10] Cornell M, McIntyre J, Myer L. Men and antiretroviral therapy in Africa: our blind spot. Trop Med Int Health. 2011;16:828-9 21418449 10.1111/j.1365-3156.2011.02767.x PMC3749374

[11] European Commission [Internet]. The state of men’s health in Europe report. Brussels: European Union; 2011. Available from: http://ec.europa.eu/health/population_groups/docs/men_health_report_en.pdf[cited 2014 June 6].

[12] Centers for Disease Control and Prevention. Workers Memorial Day – April 28, 2012. MMWR Morb Mortal Wkly Rep. 2012;61:28122534760

[13] McKinlay E, Kljakovic M, McBain L. New Zealand men's health care: are we meeting the needs of men in general practice? J Primary Health Care 2009;1(4):302-10 20690339

[14] Barker G, Ricardo C, Nascimento M, Olukoya A, Santos C. Questioning gender norms with men to improve health outcomes: evidence of impact. Glob Public Health. 2010;5:539-53 19513910 10.1080/17441690902942464

[15] Juel K, Christensen K. Are men seeking medical advice too late? Contacts to general practitioners and hospital admissions in Denmark 2005. J Public Health (Oxf). 2008;30:111-3 17981887 10.1093/pubmed/fdm072

[16] Brott A, Dougherty A, Williams ST, Matope JH, Fadich A, Taddelle M. The economic burden shouldered by public and private entities as a consequence of health disparities between men and women. Am J Mens Health. 2011;5:528-39 22064880 10.1177/1557988311421214

[17] White A, McKee M, Richardson N, de Visser R, Madsen SA, de Sousa BC. Europe’s men need their own health strategy. BMJ. 2011;343:d739 10.1136/bmj.d7397 22127516

[18] Hunt K, Wyke S, Gray CM, Anderson AS, Brady A, Bunn C, et al. A gender-sensitised weight loss and healthy living programme for overweight and obese men delivered by Scottish Premier League football clubs (FFIT): a pragmatic randomised controlled trial. Lancet. 2014;21 Jan 10.1016/S0140-6736(13)62420-4 PMC4524002 24457205

[19] Brugha RF, Kevany JP, Swan AV. An investigation of the role of fathers in immunization uptake. Int J Epidemiol. 1996;25:840-5 8921465 10.1093/ije/25.4.840

[20] Lindegren ML, Kennedy CE, Bain-Brickley D, Azman H, Creanga AA, Butler LM, et al. Integration of HIV/AIDS services with maternal, neonatal and child health, nutrition, and family planning services. Cochrane Database Syst Rev. 2012;9:CD010119 22972150 10.1002/14651858.CD010119 PMC12551653

[21] Dworkin SL, Treves-Kagan S, Lippman SA. Gender-transformative interventions to reduce HIV risks and violence with heterosexually active men: a review of the global evidence. AIDS Behav. 2013;17:2845-63 23934267 10.1007/s10461-013-0565-2

[22] World Health Organization, Regional Office for Europe [Internet]. Men’s Health. Geneva: WHO; 2014. Available from: http://www.euro.who.int/en/health-topics/health-determinants/gender/activities/mens-health [cited 2014 Feb 21].

[23] Hagedoorn M, Sanderman R, Ranchor AV, Brilman EI, Kempen GI, Ormel J. Chronic disease in elderly couples: are women more responsive to their spouses' health condition than men? J Psychosom Res. 2001;51:693-6 11728511 10.1016/s0022-3999(01)00279-3

