# Corrigendum

**DOI:** 10.2471/BLT.14.100914

**Published:** 2014-09-01

In Volume 92, Issue 8, August 2014, page 620, the first sentence of the first paragraph under “Competing interests” should begin: “PB reports an honorarium from Eli Lilly and Company to conduct a training session on men’s health, …”.

